# *Drosophila melanogaster* as a model to study drug addiction

**DOI:** 10.1007/s00439-012-1146-6

**Published:** 2012-02-17

**Authors:** Karla R. Kaun, Anita V. Devineni, Ulrike Heberlein

**Affiliations:** 1 Department of Anatomy, University of California San Francisco, 1550 4th St, MC2822, San Francisco, CA 94158 USA; 2 Present Address: HHMI Janelia Farm Research Campus, 19700 Helix Drive, Ashburn, VA 20147 USA

## Abstract

Animal studies have been instrumental in providing knowledge about the molecular and neural mechanisms underlying drug addiction. Recently, the fruit fly *Drosophila* *melanogaster* has become a valuable system to model not only the acute stimulating and sedating effects of drugs but also their more complex rewarding properties. In this review, we describe the advantages of using the fly to study drug-related behavior, provide a brief overview of the behavioral assays used, and review the molecular mechanisms and neural circuits underlying drug-induced behavior in flies. Many of these mechanisms have been validated in mammals, suggesting that the fly is a useful model to understand the mechanisms underlying addiction.

## Introduction

Drug addiction is a disorder characterized by excessive use of a drug to the point of compulsive drug seeking and consumption. The American Psychiatric Association (DMS-IV) differentiates between substance abuse, considered an earlier stage of addiction, and substance dependence. Substance abuse is defined as continued drug use despite interpersonal problems, legal problems, failure to fulfill obligations, or physically hazardous situations. The criteria for substance dependence include physical symptoms, such as tolerance and withdrawal, as well as signs of uncontrolled use, which include giving up normal activities and continued use despite knowledge of self-harm and the desire to stop. These definitions highlight the fact that addiction is an exclusively human phenomenon. However, animal models have been used to study specific aspects of addiction, and have proved invaluable in understanding the underlying neural and molecular mechanisms.

Animal models allow the experimenter to focus on distinct components of the addiction process, ranging from simple, acute drug responses to more complex behaviors such as drug seeking, self-administration, and relapse. Each behavioral model has advantages and disadvantages. Whereas the more complex models likely have greater relevance to the human condition, assays for acute drug responses are simpler to perform and thus provide the potential for high-throughput analysis, facilitating the identification of the underlying mechanisms.

Although rodent models have provided crucial insights into the mechanisms underlying drug-related behaviors, they are not ideal for unbiased, forward genetic approaches aimed at identifying novel and unsuspected mechanisms. This is due primarily to the expense and time required for animal maintenance, breeding, and behavioral analyses. In contrast, the fruit fly *Drosophila melanogaster* is one of the most genetically and experimentally accessible model organisms in biology. In this review the terms *Drosophila* and flies will refer exclusively to this species. While for many years flies were used primarily to identify the molecular and neural mechanisms regulating acute drug responses, the recent development of assays that measure drug self-administration and reward has allowed the analysis of these more complex behaviors.

## *Drosophila* as a model system to study behavior

*Drosophila* has been used to gain insight into molecular, cellular, developmental, and disease processes that are conserved in mammals, including humans, as most of these fundamental biological mechanisms are shared throughout the animal kingdom. Although mammals have two to three times as many genes as flies, they have approximately the same number of gene families (Holland 2003). About 75% of human disease genes have related sequences in *Drosophila,* suggesting that flies can serve as an effective model to study the function of a wide array of genes involved in human disease (Adams et al. 2000; Reiter et al. 2001). The nervous system of the fly comprises approximately 300,000 neurons including a brain, ventral nerve cord (the equivalent of the spinal cord), and peripheral nervous system. Despite their relatively small number of neurons in comparison with mammals, flies exhibit many complex behaviors such as associative learning, sensorimotor integration, and social behaviors (Quinn et al. 1974; Pick and Strauss 2005; Greenspan and Ferveur 2000; Chen et al. 2002).

The classical advantages of using *Drosophila* include factors such as cost, size, fecundity, and timescale. First, flies are easy and inexpensive to rear in the laboratory using small vials or bottles and a yeast-based food medium. Due to their small size, thousands of genotypes of flies can be maintained in a typical laboratory. Second, due to their high fecundity, hundreds of flies can be obtained from a single female. Third, flies have a rapid life cycle, requiring only 10 days at 25°C to develop from egg to mature adult.

For these reasons, flies have long represented an ideal organism to conduct mutagenesis screens to isolate genes regulating a particular biological process of interest (“forward genetics”, i.e. going from phenotype to gene). The advent of genetic transformation in the 1980s also allowed for “reverse genetics” (i.e. going from gene to phenotype) by allowing researchers to introduce specific genes of interest into a fly (Rubin and Spradling 1982). The subsequent sequencing and annotation of the *Drosophila* genome have greatly facilitated both of these approaches (Adams et al. 2000).

In recent years, the generation of large collections of publicly available mutants and other transgenic tools has allowed for the functional study of nearly any fly gene of interest. The traditional use of X-ray or chemical mutagenesis is becoming gradually supplanted by insertional mutagenesis, in which a transposable genetic element creates a mutation by inserting into a random genomic site, and the gene affected can be easily identified by sequencing the flanking DNA (Bingham et al. 1981). Several groups have now generated large mutant collections for which the insertion site in each mutant has been sequenced (Bellen et al. 2004; Thibault et al. 2004; Schuldiner et al. 2008). In addition, an RNA interference (RNAi) library has been generated in which each fly line contains an inducible RNAi construct for silencing a single fly gene, with nearly 90% of the fly genome represented (Dietzl et al. 2007).

**Fig. 1:**
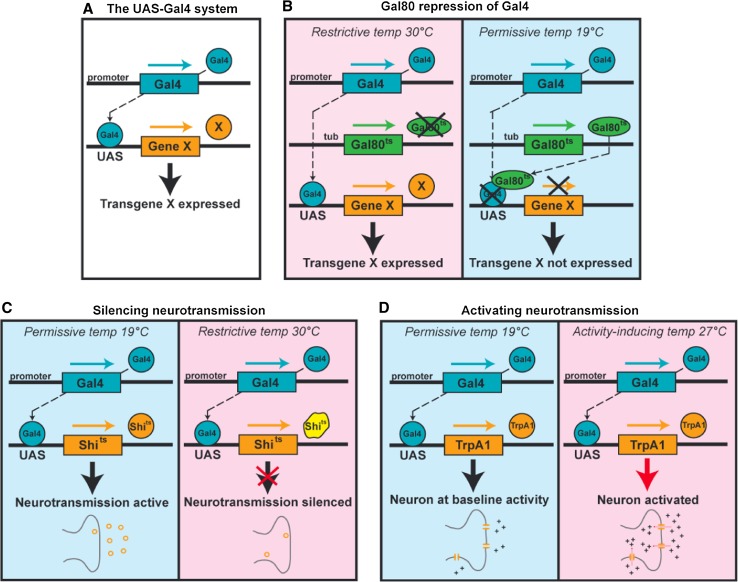
Common genetic tools in *Drosophila.* **a** The Gal4/UAS system (Brand and Perrimon 1993). The transcriptional activator Gal4 is expressed in a spatially restricted pattern and activates any gene placed downstream of the upstream activating sequence (UAS). **b** The TARGET system (McGuire et al. 2003). At the restrictive temperature (30°C), Gal80^ts^ is inactive, Gal4 is active and UAS-driven genes are expressed. At the permissive temperature (19°C), Gal80^ts^ is active, Gal4 is inhibited, and UAS-driven genes are not expressed. **c** The Shibire^ts^ system (Kitamoto 2001). At the restrictive temperature (30°C), but not the permissive temperature (19°C), Shi^ts^ blocks neurotransmission by disrupting endocytosis and thereby depleting synaptic vesicles. **d** The TrpA1 system (Hamada et al. 2008; Pulver et al. 2009). At the restrictive temperature (27°C), but not the permissive temperature (19°C), cation flow through the temperature-gated cation channel dTRPA1 causes neuronal depolarization

Some of the genetic tools developed in *Drosophila* have particular relevance to studying the relationship between genes, the brain, and behavior. For example, genetic tools in flies allow one to manipulate the nervous system independently of other tissues in the body. Furthermore, because different neural circuits may have distinct and perhaps opposing roles in behavior, one would ideally like to target specific sets of neurons within the brain. This cellular specificity can be accomplished by the bipartite Gal4/UAS system, in which the transcriptional activator Gal4 is expressed in a spatially restricted pattern and activates any gene placed downstream of the upstream activating sequence (UAS) (Brand and Perrimon 1993; Fig. 1a). The generation and characterization of thousands of Gal4 lines expressed in various patterns allow for manipulation of specific brain regions or neuronal types (Pfeiffer et al. 2008). This technique allows one to ask in which neurons a particular gene functions to regulate a behavioral response. These patterns can be further spatially refined to very small subsets of neurons using the “split Gal4 system” in which the DNA-binding and transcriptional-activation domains of Gal4 are targeted to different neuronal subsets using different promoters; transcriptional activation of target genes occurs only in neurons expressing both domains (Luan et al. 2006). Temporal specificity can be achieved by using a temperature-sensitive Gal4 repressor called Gal80^ts^ and shifting the flies from the permissive to the restrictive temperature during a particular time period (McGuire et al. 2003; Fig. 1b).

In addition to studying the function of genes within the nervous system, the Gal4/UAS system is well suited to studying neural circuit function. UAS lines are available in which proteins that inducibly control neuronal activity can be expressed, allowing one to activate or silence a particular set of neurons during a specific behavioral task. For example, neurons can be silenced using Shibire^ts^, a temperature-sensitive dynamin allele that blocks synaptic vesicle recycling (Fig. 1c), and neurons can be activated using TrpA1, a temperature-sensitive cation channel that causes neuronal depolarization (Fig. 1d) (Kitamoto 2001; Hamada et al. 2008). Furthermore, the development of a second binary system in addition to the Gal4/UAS system, the LexA/LexAop system (Lai and Lee 2006), allows for the independent manipulation of multiple neural circuits, such as activating some neurons while inhibiting others. Thus, flies have now become a leading model organism for studying not only the molecular mechanisms but also the neural circuits that underlie behavior.

## Models to study ethanol-related behaviors in flies

Ethanol is the drug that has been by far the most intensively studied in *Drosophila*, and will therefore be the main focus of this review. Ethanol is a commonly abused psychoactive drug that can produce both short-term behavioral impairment as well as long-term addiction. Unlike other drugs such as cocaine and nicotine, ethanol does not act on a single molecular target but instead is thought to affect a variety of molecules, including multiple ion channels (Koob 2004). Fruit flies encounter ethanol in their natural environment since one of the main metabolites in fermenting fruit is ethanol. Ethanol can act as a long-distance signal to draw flies to rotting fruit, as flies are attracted to low concentrations of ethanol vapor (Dudley 2002; Hoffmann and Parsons 1984). Female flies prefer to lay their eggs in media containing up to 5% ethanol (McKenzie and Parsons 1972), and larvae efficiently metabolize ethanol and use it as a food source (Geer et al. 1993).

Despite this long-standing relationship between *Drosophila* and ethanol, the molecular underpinnings of the effects of ethanol on fly behavior were not investigated until relatively recently. Several types of ethanol-related behaviors have now been characterized in flies, with the goal of using the abundant genetic tools in *Drosophila* to understand the underlying mechanisms. These behaviors range from simple to complex: (1) acute locomotor responses to ethanol, (2) ethanol tolerance following an initial exposure, and (3) ethanol preference and conditioned preference behaviors that model specific facets of addiction.

### Acute ethanol sensitivity

Flies exhibit acute responses to ethanol exposure that are quite similar to those of mammals, including humans (Morean and Corbin 2010). There is evidence in humans as well as mammalian models that sensitivity to acute ethanol-induced motor impairment correlates inversely with ethanol consumption and risk of abuse, and that the same genes can influence both types of behavior (Schuckit 1994; Morean and Corbin 2010; Kurtz et al. 1996; Thiele et al. 1998; Hodge et al. 1999). Studying these simpler ethanol responses, which are often easier to test in the laboratory, is therefore likely to provide insight into the mechanisms regulating more complex addiction-related behaviors as well.

To measure acute ethanol responses in flies, ethanol is typically administered in the form of pure ethanol vapor mixed with air at a specified ratio, allowing one to control the ethanol concentration that the flies receive (Wolf et al. 2002). Ethanol can also be administered to flies by injection, though few studies have employed this technique (Dzitoyeva et al. 2003). Low to moderate concentrations of ethanol induce locomotor hyperactivity, which can be measured by filming the flies and using tracking software to identify the flies and calculate their locomotor speed (Wolf et al. 2002).

**Fig. 2:**
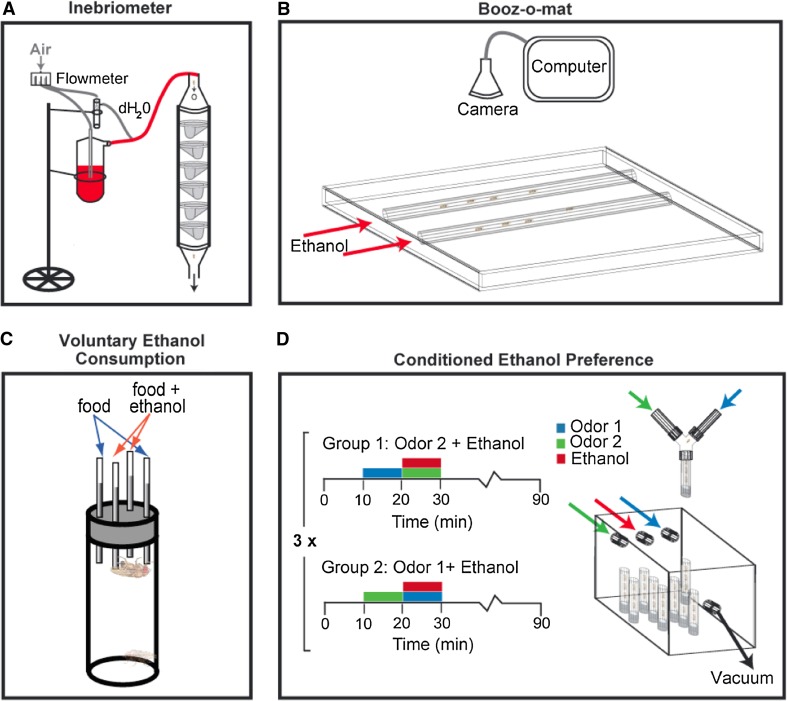
Assays to measure alcohol-induced behavior in *Drosophila*. **a** The inebriometer measures ethanol-induced loss of postural control by measuring the time required for flies to fall down the mesh baffles from the top to the bottom of the column (Weber 1988; Moore et al. 1998). **b** The booz-o-mat allows for the measurement of ethanol-induced hyperactivity and sedation while streaming vaporized ethanol into horizontal tubes containing groups of flies. Hyperactivity is measured by filming the flies and using tracking software to calculate their locomotor speed. Sedation is measured by recording the time required for flies to exhibit the loss-of-righting reflex (Wolf et al. 2002). **c** The two-choice CAFE assay measures consumption preference for food containing ethanol compared to normal food (Ja et al. 2007; Devineni and Heberlein 2009). **d** Conditioned ethanol preference is measured by training the flies in a sealed container to associate a neutral odor with the presence of an intoxicating dose of ethanol, and later testing preference for that odor in the absence of ethanol using a Y-maze (Kaun et al. 2011)

In contrast, high concentrations of ethanol elicit loss of postural control and eventually sedation (Moore et al. 1998; Rothenfluh et al. 2006; Corl et al. 2009). Loss of postural control was initially assayed in the inebriometer, a vertical column containing mesh baffles (Weber 1988; Cohan and Graf 1985; Moore et al. 1998; Fig. 2a). Flies naturally exhibit negative geotaxis and therefore tend to remain at the top of the column, but as they lose postural control they gradually fall from one baffle to the next. Ethanol sensitivity can therefore be measured as the time required for the flies to reach the bottom of the column. Negative geotaxis has also been directly assayed as a measure of ethanol sensitivity by quantifying the vertical distance that flies climb after being knocked to the bottom of a vial (Bhandari et al. 2009). More recently, ethanol-induced loss of postural control (referred to more simply as “sedation”) has been assayed manually using a loss-of-righting reflex assay, in which one counts the number of flies that fail to regain upright posture after being knocked over (Fig. 2b) (Rothenfluh et al. 2006; Corl et al. 2009).

### Ethanol tolerance

In flies, as in mammals, repeated exposure to ethanol induces tolerance, which is defined as an acquired resistance to the effects of the drug. Tolerance is one of the DSM-IV criteria for alcohol dependence (American Psychiatric Association 1994) and has been correlated with heavy drinking and alcohol abuse (Schuckit et al. 2008). In flies, ethanol tolerance is assayed by measuring the decrease in sensitivity to ethanol-induced motor impairment after ethanol pre-exposure. This change in sensitivity can be assayed using the inebriometer (Scholz et al. 2000), the sedation assay (Berger et al. 2004), or negative geotaxis (Bhandari et al. 2009), and can be measured as a change in either the proportion of impaired flies (Urizar et al. 2007), latency or duration of impairment (Dzitoyeva et al. 2003; Devineni et al. 2011), or the recovery time after sedation (Berger et al. 2004; Cowmeadow et al. 2005). Ethanol tolerance appears to be robust to variations in the tolerance protocol, as the studies cited above vary significantly in the timing and concentration of ethanol exposure.

Two types of ethanol tolerance have been characterized in flies, termed rapid and chronic. Rapid tolerance is induced by relatively brief exposure to a sedating concentration of ethanol, while chronic tolerance is induced by prolonged (~24 h) exposure to a low ethanol concentration that does not produce overt intoxication (Berger et al. 2004). Rapid and chronic tolerance are mediated by distinct mechanisms; for example, only chronic tolerance is dependent on protein synthesis (Berger et al. 2004).

### Voluntary ethanol consumption

The behavioral assays described above were designed to study relatively simple behaviors associated with ethanol exposure, and much has been learned from these assays regarding the molecular mechanisms underlying the effects of ethanol. However, in order to relate these discoveries to addiction-related processes, more complex assays that more closely mimic mammalian behavior are needed. A recently developed ethanol self-administration assay demonstrates that flies prefer to consume ethanol-containing food over regular food and that this preference exhibits several features reminiscent of compulsive alcohol consumption (Ja et al. 2007; Devineni and Heberlein 2009).

Ethanol consumption can be measured in flies using a two-choice assay similar to the two bottle choice assay used in rodent studies (Fig. 2c) (Devineni and Heberlein 2009). Flies exhibit a robust, dose-dependent preference for food containing 5–25% ethanol. This ethanol preference cannot be entirely explained by either chemosensory or caloric attraction to ethanol; ethanol preference persists in the absence of olfactory or gustatory input, and preference is not altered by varying the relative caloric content of the solutions (Devineni and Heberlein 2009). Flies also show increased ethanol consumption over time, and, when food deprived, voluntarily consume ethanol to pharmacologically relevant concentrations. Importantly, flies demonstrate two additional criteria of addiction-like behavior; they will overcome an aversive stimulus, the bitter-tasting compound quinine, in order to consume ethanol, and they rapidly return to high levels of ethanol consumption after ethanol deprivation, modeling a relapse-like effect (Devineni and Heberlein 2009).

### Conditioned preference for ethanol reward

To test directly whether intoxicating doses of ethanol are rewarding to flies, a conditioned ethanol preference assay was recently developed (Kaun et al. 2011; Fig. 2d). In this assay, flies are initially exposed to two neutral odor cues, one of which is paired with a moderately intoxicating exposure to ethanol vapor. Flies are later offered a choice between the two odors, and preference for the ethanol-associated odor is measured. Similar to mammalian conditioned place preference (CPP) models, this assay uses conditioned preference to assess the rewarding properties of ethanol intoxication. This assay has some advantages over the ethanol self-administration assay: (1) the ethanol concentration that the flies experience can be controlled by the experimenter and (2) the ethanol stimulus is removed during the test, allowing measurement of the rewarding value of the drug rather than immediate preference for the drug.

When flies have been trained to associate an odor cue with ethanol intoxication, they show initial aversion to the cue, which, within 12–15 h, transforms into a long-lasting preference (Kaun et al. 2011). The development of conditioned preference is dependent on the ethanol concentration; preference is induced only by exposure to moderate ethanol doses that induce locomotor hyperactivity. Conditioned preference is not induced by lower ethanol concentrations that fail to elicit behavioral changes or higher concentrations that cause sedation. Thus, flies seem to require an intoxicating, but not sedating, dose of ethanol for it to be remembered as rewarding. Remarkably, flies will endure electric shock in order to attain the cue associated with ethanol, indicating that they are willing to tolerate punishment to seek the drug (or, in this case, a cue that predicts the presence of the drug) (Kaun et al. 2011). This response is reminiscent of compulsive behavior such as impaired response inhibition observed in mammalian studies of drug reward. Furthermore, flies will endure a stronger shock intensity to attain a cue associated with ethanol than a cue associated with sugar, suggesting that the preference for ethanol is distinct from a preference for food reward (Kaun et al. 2011).

## Molecular mechanisms underlying ethanol-induced behavior in *Drosophila*

As described above, an array of assays has been established to study various aspects of ethanol-induced behavior in *Drosophila*. All of these assays are relatively simple, robust, and high-throughput, allowing researchers to conduct forward genetic screens to identify the underlying mechanisms. The genes identified in these screens have led to the characterization of diverse molecular and cellular processes that mediate ethanol-induced behavior in flies.

### Molecular pathways

Several classical molecular signaling pathways have been implicated in regulating sensitivity to ethanol-induced motor impairment in flies, including the epidermal growth factor receptor (EGFR), phosphoinositide 3-kinase (PI3K)/Akt, and cyclic adenosine monophosphate (cAMP) pathways. Genetic and pharmacological evidence indicates that the EGFR and cAMP pathways promote resistance to ethanol sedation (Corl et al. 2009; Moore et al. 1998), while the PI3K/Akt pathway enhances ethanol sedation (Eddison et al. 2011). However, these pathways are likely to regulate ethanol sensitivity in more complex ways depending on factors such as the cell types in which they are expressed and the presence of multiple protein isoforms. For example, a mutation disrupting the function of the type II regulatory subunit of protein kinase A (PKA), a key effector of cAMP signaling, causes the opposite effect on ethanol sedation as expected from previous manipulations that disrupt overall PKA signaling (Park et al. 2000; Moore et al. 1998).

A genetic screen revealed that *scabrous* (*sca*), encoding a secreted protein that negatively regulates the Notch signaling pathway (Baker et al. 1990; Powell et al. 2001), is required for ethanol reward memory. Notch signaling is required for long-term memory formation in flies, suggesting that *sca* may contribute generally to long-term memory processes (Presente et al. 2004; Ge et al. 2004). However, a mutation of *sca* that affects ethanol reward memory does not affect short-term memory (LaFerriere et al. 2008). Notch signaling has been shown to regulate migration, morphology, synaptic plasticity and survival of immature and mature neurons (Ables et al. 2011). It will be interesting to examine how *sca* and the Notch pathway affect the neural plasticity underlying memory for ethanol reward.

Transcription and translation have been implicated in ethanol-induced behaviors. Two genes encoding putative transcriptional regulators, *Drosophila LIM*-*domain only* (*dLmo*) and *hangover* (*hang*), were identified as regulators of ethanol sedation and ethanol tolerance, respectively (Lasek et al. 2011a; Scholz et al. 2005). One likely target gene whose expression is regulated by *dLmo*, the *Drosophila* homolog of *anaplastic lymphoma kinase* (*dAlk*), has been identified and shown to regulate ethanol sedation (Lasek et al. 2011b).

A mutation in *krasavietz* (*kra*), which encodes a predicted translation initiation factor that inhibits protein translation in vitro (Lee et al. 2007), causes decreased sensitivity to ethanol-induced sedation, decreased rapid and chronic ethanol tolerance, and decreased ethanol consumption (Berger et al. 2008; Devineni and Heberlein 2009). Although protein synthesis is required for chronic tolerance, it is not required for the development of rapid tolerance and is unlikely to occur during the brief timescale of acute ethanol intoxication (~30 min; Berger et al. 2004). However, treating flies with a protein synthesis inhibitor prior to ethanol exposure caused pronounced resistance to ethanol impairment (Berger et al. 2004), suggesting that some proteins that are constitutively synthesized in the absence of ethanol mediate naive ethanol sensitivity. The role of protein synthesis in ethanol consumption has not been directly tested.

Finally, molecular pathways involved in cellular stress responses have been implicated in ethanol tolerance. *hang* mutants, which show decreased ethanol tolerance, also show sensitivity to oxidative stress and decreased heat-ethanol cross-tolerance (i.e. tolerance to ethanol stimulated by heat shock stress instead of ethanol exposure; Scholz et al. 2005). The gene *jwa*, a retinoic acid-responsive gene whose product associates with the cytoskeleton, mediates oxidative and heat stress responses and also promotes ethanol tolerance (Li et al. 2008). Since high doses of ethanol induce cellular stress, which in some ways mimics oxidative and heat stress, it may not be surprising that common molecular pathways respond to ethanol as well as other stressors (e.g. Wu and Cederbaum 2009; Wilke et al. 1994; Piper 1995).

### Cellular mechanisms

One of the key cellular processes that has been implicated in ethanol-induced behaviors in flies is cytoskeletal dynamics. *thousand and one* (*tao*), which was identified as a critical regulator of ethanol-induced hyperactivity, implicated microtubule dynamics in the hyperactivity response (King et al. 2011). *tao* was shown to function through the conserved kinase PAR-1 (also called MARK in mammals) to regulate the microtubule-binding protein Tau during fly brain development (Matenia and Mandelkow 2009; King et al. 2011). The mouse homolog of *jwa*, which promotes ethanol tolerance in flies (see above), is also a microtubule-associated protein (Li et al. 2008; Chen et al. 2007).

In addition to microtubule organization, the regulation of actin has been implicated in ethanol responses. *Rho GTPase activator protein 18B* (*RhoGAP18B*) regulates sensitivity to both ethanol-induced sedation and hyperactivity through different protein isoforms (Rothenfluh et al. 2006). RhoGAP18B is a GTPase activating protein that regulates ethanol sensitivity by functioning through Rho family GTPases, which are key regulators of actin dynamics (Rothenfluh et al. 2006). Additionally, Kra (described above) interacts with the crosslinking protein Short stop to regulate actin organization, suggesting that that actin regulation may underlie some of its diverse effects on ethanol-induced behavior (Lee et al. 2007; Sanchez-Soriano et al. 2009). However, it is important to note that changes in neither microtubule nor actin organization have been directly linked to altered ethanol responses in these mutants.

Finally, the integrin class of cell adhesion molecules has been implicated in ethanol-induced behaviors. Mutations in the alpha-integrin gene *scab* (*scb*) or the β-integrin gene *myospheroid* (*mys*) cause increased ethanol sensitivity as well as increased tolerance (Bhandari et al. 2009). It will be interesting to determine the mechanisms by which disruptions in cytoskeletal organization or cell adhesion lead to altered ethanol responses.

### Synaptic function and neuronal excitability

Synapse number has recently been implicated in ethanol sensitivity in flies. Several genetic manipulations that lead to increased ethanol sedation sensitivity, such as mutations in *arouser* (*aru*) or *amnesiac* (*amn*) and overexpression of *PI3K* or *Ras homolog enriched in brain* (*Rheb*), also increase synapse number at the larval neuromuscular junction (NMJ) and/or the adult central brain (Eddison et al. 2011). An environmental manipulation, adult social isolation, which is known to reduce the number of synapses of a specific set of fly brain neurons (Donlea et al. 2009), also reduces ethanol sensitivity (Eddison et al. 2011). Furthermore, social isolation concurrently restores normal synapse number and ethanol sensitivity to the ethanol-sensitive mutant *aru* (Eddison et al. 2011). This correlation between synapse number and ethanol sedation sensitivity using multiple independent genetic and environmental manipulations suggests that increased synapse number may directly promote increased sensitivity to ethanol sedation.

In addition to providing a novel cellular mechanism by which ethanol behaviors can be regulated, these findings suggest the hypothesis that ethanol tolerance may result from a compensatory decrease in synapse number induced by the initial ethanol exposure. While this hypothesis has not been directly tested, *hang*, which promotes ethanol tolerance (see above), negatively regulates synapse number at the larval NMJ (Schwenkert et al. 2008). For both ethanol sensitivity and tolerance, it remains to be investigated whether increased synapse number translates into increased postsynaptic excitation (or inhibition), and whether ethanol sensitivity depends on increased synapse number generally throughout the nervous system or in specific neurons.

There is abundant evidence that synaptic transmission regulates ethanol-induced behaviors in flies. Flies carrying a mutation in *Syntaxin 1A* (*Syx1A*) or *shibire* (*shi,* encoding *Drosophila* dynamin), which are, respectively, required for synaptic vesicle docking and recycling, show defects in ethanol sedation tolerance (Krishnan et al. 2011). Use of conditional mutations revealed that normal synaptic vesicle release is required immediately after initial ethanol exposure rather than after recovery from intoxication to promote tolerance (Krishnan et al. 2011). A different study showed that flies lacking *Synapsin* (*Syn*), encoding a presynaptic vesicle scaffolding protein, unexpectedly show increased ethanol tolerance (Godenschwege et al. 2004). This result may reflect the fact that Synapsin is involved not only in regulating neurotransmitter release but also in neurite growth, synaptic formation and maturation, and in segregating the reserve and readily releasable pools of vesicles (Cesca et al. 2010). Finally, Homer, a protein that interacts with postsynaptic scaffolding and signaling proteins, including metabotropic glutamate receptors, regulates both initial ethanol sensitivity and ethanol tolerance (Urizar et al. 2007).

The major regulators of neuronal excitability that have been implicated in ethanol-induced behaviors in flies are the γ-aminobutyric acid B (GABA_B_) receptors and the large conductance calcium-activated potassium (BK) channels. GABA_B_ receptor activity promotes sensitivity to ethanol sedation but reduces rapid ethanol tolerance (Dzitoyeva et al. 2003). As in mammals, *Drosophila* GABA_B_ receptors are metabotropically coupled to potassium channels, thereby inhibiting neuronal excitability due to potassium efflux (Mezler et al. 2001). The BK channel encoded by the gene *slowpoke* (*slo*) has also been implicated in rapid ethanol tolerance, but in the opposite direction. Expression of the fly BK channel is upregulated by ethanol exposure and its function is required for the development of rapid ethanol tolerance; induction of BK channel expression is in fact sufficient to induce ethanol resistance, mimicking the tolerant state (Cowmeadow et al. 2005, 2006). The fact that GABA_B_ receptors and BK channels likely affect neuronal excitability in the same direction, but regulate tolerance in opposite ways, suggests that they may function in different subsets of neurons that exert opposing effects on behavior. Alternatively, it has been proposed that BK channels may in fact enhance neuronal excitability by reducing the refractory period or enhancing firing rates, allowing neurons to compensate for the depressant effect of ethanol during sedation (Atkinson 2009).

In addition to classical neurotransmitters such as GABA, neuromodulators, including biogenic amines and neuropeptides, also regulate ethanol-induced behavior in flies. Dopamine promotes ethanol hyperactivity through the D1-like receptor DopR (Bainton et al. 2000; Kong et al. 2010b) and is also required for conditioned ethanol preference (Kaun et al. 2011). Octopamine, a biogenic amine thought to be the invertebrate analog of norepinephrine, is essential for the development of rapid but not chronic ethanol tolerance (Scholz 2000; Berger et al. 2004). Two neuropeptides produced in the fly brain, neuropeptide F (NPF) and insulin, have been shown to regulate ethanol sedation. NPF, the fly homolog of neuropeptide Y, enhances ethanol sedation (Wen et al. 2005). Mutations in the insulin receptor (InR) cause increased sedation sensitivity, as does overexpression of the adaptor protein p60 to inhibit the coupling between the insulin receptor (InR) and PI3K, the main effector of insulin signaling (Corl et al. 2005). These results indicate that insulin acts through PI3K to promote sedation resistance. However, a different study (discussed earlier) using several more direct manipulations of the PI3K/Akt pathway demonstrated that this pathway promotes sedation sensitivity (Eddison et al. 2011). PI3K may therefore have opposing roles in regulating ethanol sedation depending on the upstream molecule to which it is coupled and the cell type in which it is expressed. In general, the mechanisms by which these neuromodulators affect postsynaptic and/or presynaptic cells have not yet been characterized. It thus remains an open question whether they directly affect postsynaptic excitability or modulate other pre- or postsynaptic properties.

### Genome-wide studies

The majority of the genes discussed above were identified using genetic screens in which mutants exhibiting abnormal behavior were isolated. However, an alternative approach is to use transcriptional profiling to compare gene expression under different conditions. For example, one study identified genes differentially expressed in fly strains selected for increased versus decreased sensitivity to ethanol, and confirmed that mutations in many of these genes cause altered ethanol sensitivity (Morozova et al. 2007).

Three studies have identified genes whose expression is regulated by ethanol exposure, making them good candidates for mediating the development of tolerance. These studies used varying exposure protocols and collectively identified 1,669 candidate genes, 29 of which were common to all three studies and 229 of which were common to at least two out of three studies (Morozova et al. 2006; Urizar et al. 2007; Kong et al. 2010a). Many of these genes were functionally validated using mutant analysis (Morozova et al. 2006; Kong et al. 2010a), but in most cases the molecular and cellular mechanisms by which these genes function have not been determined.

## Neural circuits underlying ethanol-induced behavior in *Drosophila*

Although the neural circuits mediating ethanol-induced behaviors in *Drosophila* have not been as extensively studied as the molecular mechanisms, new tools such as Gal4 lines to target particular neurons and transgenes to manipulate neuronal activity have made the study of circuits more accessible.

In mammals, dopamine is an important regulator of many ethanol-related behaviors (Soderpalm et al. 2009). In the fly, dopamine is expressed in several clusters of neurons that project to a variety of brain regions (Nassel and Elekes 1992). As in mammals, many of these dopaminergic cells have been shown to play a role in ethanol-related behaviors. The function of dopamine in regulating ethanol hyperactivity was localized to a pair of dopaminergic neurons projecting to DopR-expressing neurons in the ellipsoid body of the central complex (Kong et al. 2010b), a region known to regulate visual and locomotor behavior, arousal, and memory (Martin et al. 1999; Wu et al. 2007; Neuser et al. 2008; Ofstad et al. 2011). The ellipsoid body is also the site of Homer function in the regulation of ethanol sedation sensitivity and tolerance (Urizar et al. 2007), though it is unknown whether Homer functions via DopR signaling, or in DopR-expressing neurons.

Dopamine neurons also mediate conditioned ethanol preference. Ethanol reward memory, like other forms of memory, can be divided into three phases: acquisition (memory formation during training), consolidation (the period between training and testing), and retrieval (expression of the memory during testing) (Krashes et al. 2007). Interestingly, silencing dopaminergic neurotransmission impairs retrieval, but not acquisition or consolidation, of ethanol reward memory (Kaun et al. 2011).

While some dopaminergic neurons innervate the ellipsoid body, others terminate in the mushroom body, a brain structure implicated in olfactory processing and learning (Davis 2011). Neurotransmission of mushroom body neurons is required for both ethanol-induced hyperactivity and conditioned ethanol preference (King et al. 2011; Kaun et al. 2011). Both behaviors are mediated by neurons in specific subregions within this structure, and distinct phases of conditioned ethanol preference are in fact localized to different mushroom body neurons (King et al. 2011; Kaun et al. 2011). Together, these studies demonstrate that different ethanol-induced behaviors can be mapped to distinct neural loci, and that some brain structures, such as the mushroom body, are important for multiple behaviors.

## Mammalian validation of mechanisms underlying ethanol-induced behavior

Now that years of research have implicated many different molecular and cellular pathways in mediating fly responses to ethanol, it is important to ask whether these mechanisms function in mammals as well. In fact, many of the genes and molecular pathways implicated in *Drosophila* ethanol responses play a similar role in mammals (see Table 1). For example, the cAMP, EGFR, and NPF/NPY pathways all regulate ethanol sensitivity similarly in flies and rodents (Moore et al. 1998; Wand et al. 2001; Corl et al. 2009; Wen et al. 2005; Thiele et al. 1998). Furthermore, these pathways regulate not only ethanol sensitivity but also ethanol consumption in rodents (Wand et al. 2001; Corl et al. 2009; Thiele et al. 1998). Thus, simple behavioral assays that are readily used for genetic screening in flies can yield candidate genes that have homologous roles in rodent models. Moreover, an FDA-approved drug that inhibits the function of EGFR, a molecule first shown to regulate ethanol-related behavior in the fly, has been shown to be effective in a preclinical rat model of ethanol addiction (Corl et al. 2009).

**Table 1:** Selected genes mediating ethanol-induced behaviors in flies

Gene	Mechanism of action	Ethanol-related phenotype	Reference	Homolog validated in mammals
*amn*	cAMP pathway	Increased motor impairment	Moore et al. (1998)	Wand et al. (2001)
*hppy*	Inhibits EGFR pathway	Decreased sedation	Corl et al. (2009)	
*Egfr*	EGFR/Erk pathway	Increased sedation	Corl et al. (2009)	Corl et al. (2009)
*aru*	EGFR and PI3K/Akt pathways; regulation of synapse number	Increased sedation	Eddison et al. (2011)	
*Rheb*	Tor pathway; regulation of synapse number	Increased sedation (upon overexpression)	Eddison et al. (2011)	
*sca*	Notch pathway?	Decreased conditioned preference	Kaun et al. (2011)	
*dLmo*	Transcriptional regulation of *dAlk*?	Increased sedation	Lasek et al. (2011a)	Lasek et al. (2011a)
*dAlk*	Receptor tyrosine kinase signaling	Decreased sedation	Lasek et al. (2011b)	Lasek et al. (2011b)
*hang*	Stress pathway; regulation of synapse number?	Decreased tolerance	Scholz et al. (2005)	Riley et al. (2006)
*jwa*	Stress pathway; regulation of microtubules?	Decreased tolerance	Li et al. (2008)	
*kra*	Regulation of translation? Actin regulation?	Decreased sedation; decreased tolerance; decreased ethanol consumption	Berger et al. (2008) and Devineni and Heberlein ( 2009)	
*tao*	Regulation of Tau/microtubules through par-1	Decreased hyperactivity	King et al. (2011)	
*RhoGAP18B*	Regulation of Rho family GTPases; actin regulation?	Decreased sedation	Rothenfluh et al. (2006)	
*scb*	Integrin/cell adhesion	Increased motor impairment; increased tolerance	Bhandari et al. (2009)	
*mys*	Integrin/cell adhesion	Increased motor impairment; increased tolerance	Bhandari et al. (2009)	
*Syx1A*	Synaptic transmission	Decreased tolerance	Krishnan et al. (2011)	
*shi*	Synaptic transmission	Decreased tolerance	Krishnan et al. (2011)	
*Syn*	Synaptic transmission	Increased tolerance	Godenschwege et al. (2004)	
*homer*	Postsynaptic signaling	Increased sedation; decreased tolerance	Urizar et al. (2007)	Szumlinski et al. (2005)
*GABA*-*B*-*R1*	GABA signaling	Decreased sedation	Dzitoyeva et al. (2003)	Zaleski et al. (2001)
*slo*	Calcium-activated potassium channel activity	Decreased tolerance	Cowmeadow et al. (2005)	Knott et al. (2002)
*ple*	Dopamine synthesis	Decreased hyperactivity	Bainton et al. (2000)	Friedhoff and Miller (1973)
*DopR*	Dopamine signaling	Decreased hyperactivity	Kong et al. (2010b)	El Ghundi et al. (1998)
*Tbh*	Octopamine synthesis	Decreased tolerance	Scholz et al. (2000) and Berger et al. (2004)	Tabakoff and Ritzmann (1977)
*InR*	Insulin signaling	Increased sedation	Corl et al. (2005)	
*npf*	NPF signaling	Decreased sedation	Wen et al. (2005)	Thiele et al. (1998)

This table includes the genes referred to in the text, which represent many of the genes that have been functionally characterized as regulators of ethanol-induced behavior. We have not included every gene that has been identified, but we have made an effort to include representative genes for each signaling pathway or general mechanism. Studies identifying a large number of genes with limited characterization of mechanism (e.g. Berger et al. 2008) have not been included. In cases where many genes in the same signaling pathway have been implicated, the gene initially identified is listed and the signaling pathway is described in the second column (e.g. *aru*, PI3K/Akt pathway). Unless otherwise specified, the ethanol-related phenotype described in the third column refers to the phenotype upon impairing the function of the gene product by mutation, RNAi, or pharmacology

While most of the genes affecting ethanol-induced behavior in flies have not yet been tested for a role in humans, a few have already been associated with human ethanol-related behavior. Polymorphisms in the human *ALK* gene are correlated with multiple measures of ethanol sensitivity (Lasek et al. 2011b), and polymorphisms in one human homolog of *hang*, *ZNF699*, were found to be associated with alcohol dependence (Riley et al. 2006). Recently, a genome-wide meta-analysis revealed that polymorphisms in *autism susceptibility candidate 2* (*AUTS2*) are associated with alcohol consumption (Schumann et al. 2011). Mice selected for high versus low alcohol consumption differ in expression of *AUTS2*, and downregulation of the fly homolog of *AUTS2* leads to reduced ethanol sensitivity (Schumann et al. 2011). Given the significant conservation of genes affecting ethanol responses in flies and rodents, it is likely that additional genes identified in flies will be validated in rodents and humans, and vice versa.

In addition to molecular pathways, some of the cellular mechanisms implicated in *Drosophila* ethanol responses have also been studied in mammals. For example, the role of synapse function in ethanol-induced behavior is still an emerging field of study in flies, while the effects of ethanol at the synapse have been well studied in mammals. Ethanol acts on a variety of postsynaptic receptors, most notably GABA_A_ and *N*-methyl-d-aspartic acid (NMDA) receptors, and also exerts presynaptic effects on neurotransmitter release (Siggins et al. 2005). Whether changes in synapse number are associated with altered ethanol behaviors in mammals, as is the case in flies (Eddison et al. 2011), has not yet been studied.

While at the molecular level flies and mammals share many features (Littleton and Ganetzky 2000; Lloyd et al. 2000), the anatomical organization of fly and mammalian nervous systems is quite distinct. It is therefore difficult to draw parallels between the neural circuits that regulate ethanol-induced behavior in flies and mammals. In flies, brain structures such as the ellipsoid body and the mushroom body have been implicated in various ethanol responses; it is unclear what the equivalent structures are in the mammalian brain. Nevertheless, certain conserved neurochemical systems function similarly in flies and mammals. The mammalian mesolimbic dopamine pathway, including its target regions, is perhaps the most intensely studied neural circuit in the context of alcohol reward and addiction (Soderpalm et al. 2009). Dopamine neurons were similarly found to be required for ethanol hyperactivity and reward in *Drosophila* (Kong et al. 2010b; Kaun et al. 2011). Neuropeptidergic systems, such as the NPY/NPF system, also regulate ethanol responses similarly in flies and rodents, as discussed above. Thus, the functions of neurochemically defined neural pathways, rather than morphologically defined brain regions, are likely to be conserved in regulating ethanol behaviors.

## Study of other drugs of abuse in *Drosophila*

Drugs of abuse other than ethanol have not yet been studied extensively in flies. This may be due in part to the fact that ethanol, in vapor or liquid form, can be delivered to flies quite readily and in a reproducible manner; the same is not true for drugs such as cocaine and nicotine (McClung and Hirsh 1998; Bainton et al. 2000). Although there are assays to measure the locomotor effects of some of these other drugs, there are currently no assays to investigate their rewarding and addiction-like properties. Nevertheless, several genes and molecular mechanisms regulating drug-induced behaviors have been discovered using simple locomotion assays (Table 2).

**Table 2:** Genes mediating drug-related behaviors in flies (excluding ethanol)

Gene	Mechanism of action	Drug-related phenotype	Reference	Homolog validated in mammals
*ple*	Dopamine synthesis	Decreased sensitivity to cocaine and nicotine (using drug inhibitor)	Bainton et al. (2000)	Pradhan (1983)
*Vmat* (*isoform A*)	Monoamine storage and release	Reduced cocaine-induced hyperactivity	Chang et al. (2006)	Brown et al. (2001)
*per, Clk, cyc,* and *dco*	Regulation of circadian rhythms	Reduced behavioral sensitization to cocaine	Andretic et al. (1999)	Abarca et al. (2002) and McClung et al. (2005)
*moody*	Development and permeability of blood–brain barrier	Increased sensitivity to cocaine	Bainton et al. (2005) and Schwabe et al. (2005)	
*loco*	Functions with *moody* in development of blood–brain barrier	Reduced sensitivity to cocaine	Bainton et al. (2005) and Schwabe et al. (2005)	Bishop et al. (2002) and Schwendt et al. (2007)
*RhoGAP18B*	Regulates actin cytoskeleton?	Reduced sensitivity to cocaine and nicotine	Rothenfluh et al. (2006)	
*dLmo*	Regulates dopamine receptor expression?	Increased sensitivity to cocaine and nicotine	Tsai et al. (2004)	Lasek et al. (2010)
*tao*	Mushroom body development; regulates microtubules?	Reduced sensitivity to cocaine and nicotine	King et al. (2011)	

The drug-related phenotype refers to the phenotype upon impairing the function of the gene product

### Cocaine

Cocaine is an addictive psychostimulant that causes enhanced locomotor activity and stereotypy (repetitive behavior) in mammals (Satel et al. 1991). When flies are exposed to volatilized cocaine, they show similar behavioral effects including continuous grooming at low doses, circling and aberrant walking behavior at intermediate doses, and fast, uncontrolled movements followed by body tremors and akinesia at high doses (McClung and Hirsh 1998). Repeated cocaine exposure causes flies to become increasingly sensitive to the behavioral effects of the drug, a process referred to as sensitization (McClung and Hirsh 1998). In addition to direct observation, other assays have been developed for greater control of drug delivery and simpler behavioral analysis. For example, the “crackometer” quantifies the loss of negative geotaxis and positive phototaxis (two robust innate behaviors in flies) under the influence of volatilized cocaine (Bainton et al. 2000). Semi-automation of this assay allows for high-throughput behavioral analysis (George et al. 2005). Finally, locomotor tracking systems allow for quantification of locomotor speed and pattern (Bainton et al. 2000; Dimitrijevic et al. 2004).

Unlike ethanol, cocaine acts primarily on a single class of molecular targets: it inhibits monoamine transporters, thereby increasing synaptic levels of monoamines including dopamine, serotonin, epinephrine, and norepinephrine. Inhibition of the dopamine transporter (DAT), in particular, is largely responsible for cocaine-induced locomotor hyperactivity in mammals (Giros et al. 1996). It is therefore not surprising that dopamine signaling is also required for cocaine-induced hyperactivity in flies. A key role for the dopaminergic system in mediating the effect of cocaine has been demonstrated through both pharmacological and genetic methods. Pharmacological reduction of dopamine levels or dopamine receptor function causes decreased locomotor hyperactivation by cocaine, suggesting that cocaine induces hyperactivity by increasing dopaminergic transmission (Bainton et al. 2000; Torres and Horowitz 1998; Yellman et al. 1998). However, genetic studies provide conflicting results: (1) overexpression of one isoform of the vesicular monoamine transporter (VMAT-A), which is expected to increase dopamine levels, decreases cocaine sensitivity (Chang et al. 2006), and (2) constitutive inhibition of dopaminergic transmission, predicted to block the effects of cocaine, causes cocaine hypersensitivity (Li et al. 2000). In both cases these initially counterintuitive results could be explained by compensatory adaptations in postsynaptic dopamine signaling (Chang et al. 2006; Li et al. 2000).

*Drosophila* studies have identified unanticipated genes and pathways regulating cocaine-induced behavior. For example, mutations in the circadian genes *period* (*per*), *clock* (*Clk*), *cycle* (*cyc*)*,* and *doubletime*/*discs overgrown* (*dco*) reduce behavioral sensitization to cocaine (Andretic et al. 1999). These genes may regulate cocaine sensitization by affecting dopaminergic signaling since cocaine-treated *per* mutants, unlike cocaine-treated wild-type flies, do not increase locomotion in response to a dopamine receptor agonist (Andretic et al. 1999). Circadian genes similarly mediate cocaine-induced behaviors in mammals. The mammalian homologs of *per* have been shown to regulate cocaine sensitization and CPP in mice (Abarca et al. 2002). In addition, mice lacking a functional *Clock* gene display increased cocaine reward and dopamine neuron excitability in the midbrain (McClung et al. 2005).

*moody*, which encodes two G protein-coupled receptors (GPCRs), was identified in a genetic screen for cocaine-induced loss of negative geotaxis (Bainton et al. 2005). *moody* mutant flies show enhanced cocaine sensitivity, and *moody* was shown to function in glia to regulate blood–brain barrier permeability (Bainton et al. 2005; Schwabe et al. 2005). *loco*, which encodes a regulator of G protein signaling (RGS) that terminates GPCR signaling, functions along with *moody* to regulate blood–brain barrier permeability (Schwabe et al. 2005). As predicted from its molecular function, *loco* regulates cocaine sensitivity in the opposite direction as *moody* (Granderath et al. 1999; Bainton et al. 2005). The mammalian homolog of *loco*, RGS4, has been implicated in psychostimulant use (Bishop et al. 2002; Gold et al. 2003; Schwendt et al. 2006, 2007). These studies suggest that blood–brain barrier permeability may play an important role in drug sensitivity and potentially addiction.

Genetic screens have revealed overlap between genes that regulate ethanol and cocaine sensitivity. *RhoGAP18B,* *tao*, and *dLmo*, three genes that were identified in genetic screens and shown to regulate ethanol sensitivity (see previous section), also regulate cocaine sensitivity (Rothenfluh et al. 2006; King et al. 2011; Tsai et al. 2004). Both *RhoGAP18B* and *tao* mutants exhibit cocaine resistance, while *dLmo* mutants show increased cocaine sensitivity. *dLmo* regulation of cocaine sensitivity has been mapped to a subset of the pigment-dispersing factor (PDF) neurons (Tsai et al. 2004), the primary circadian pacemaker cells in flies (Renn et al. 1999). The function of *dLmo* has been validated in mammals, as downregulation of one of the mammalian homologs, *Lmo4*, causes increased cocaine sensitivity and sensitization in mice (Lasek et al. 2010). *Lmo4* may regulate cocaine sensitivity by acting through the dopamine D2 receptor (*Drd2*) or the GluR1 subunit of the AMPA receptor, both of which show decreased expression upon *Lmo4* downregulation (Heberlein et al. 2009).

### Amphetamines

Amphetamines are psychostimulants that, like cocaine, increase monoaminergic transmission. Amphetamines cause monoamine transporters to function in reverse, transporting these transmitters into the synapse (Koob and Nestler 1997). A small number of studies have characterized the effects of amphetamines in *Drosophila*, focusing on methamphetamine and 3,4-methylenedioxy-methamphetamine (MDMA, also known as ecstasy).

One study investigated the behavioral effects of orally administering methamphetamine to adult flies. Consistent with its stimulant effects in mammals, methamphetamine increased locomotor activity of flies, interrupted sleep, and also affected male courtship (Andretic et al. 2005). Methamphetamine administration was also associated with changes in visually evoked neural activity, suggesting that it may affect visual perception (Andretic et al. 2005). Methamphetamine showed interactions with the dopaminergic system in affecting this type of neural activity, consistent with its mode of action in mammals (Andretic et al. 2005).

In a different study, MDMA was orally administered to *Drosophila* larvae, which caused reduced feeding and locomotion (Dasari et al. 2007). It has not been determined whether MDMA also produces stimulant effects on *Drosophila* behavior. Larvae fed MDMA did not show an acute difference in levels of dopamine or serotonin, but did contain higher levels of both transmitters as adults, suggesting that monoamine synthesis may be upregulated over time in response to MDMA-induced monoamine depletion (Dasari et al. 2007).

### Nicotine

Nicotine, the major addictive component of tobacco, affects mammalian behavior by activating nicotinic acetylcholine receptors (Nestler 2005). When exposed to volatilized nicotine, flies exhibit locomotor hyperactivity and spasmodic movements leading to grooming at low doses and hypokinesis and akinesia at higher doses (Bainton et al. 2000). Similar to cocaine, nicotine exposure dose-dependently impairs negative geotaxis in flies (Bainton et al. 2000). In mammals, the addictive properties of nicotine are thought to be mediated by both direct and indirect activation of dopaminergic neurons (Nestler 2005). The locomotor effects of nicotine in flies are similarly dependent on dopamine, as pharmacological depletion of dopamine reduces nicotine sensitivity (Bainton et al. 2000). Aside from dopamine, little is known about the molecular mechanisms mediating nicotine sensitivity in flies. However, several genes known to mediate cocaine sensitivity in flies have also been shown to regulate nicotine sensitivity: *moody* mutant flies are sensitive to the effects of both drugs, whereas *RhoGAP18B* and *tao* mutants are resistant (Bainton et al. 2005; Rothenfluh et al. 2006; King et al. 2011). These genes suggest that certain shared mechanisms may regulate multiple types of drug addiction in flies.

## Conclusions and future directions

For many years flies have been used as a model to study acute drug responses, with a particular focus on ethanol, and the mechanisms underlying these behaviors have turned out to be remarkably conserved from flies to mammals. More recently, the development of new assays, in particular the voluntary ethanol consumption and conditioned ethanol preference assays, has demonstrated that flies exhibit addiction-like behavior. Flies fulfill several of the criteria proposed for an animal model of alcohol addiction (Cicero 1979; McBride and Li 1998). The ethanol consumption assay reveals that (1) flies voluntarily consume ethanol and can achieve pharmacologically relevant internal ethanol levels, (2) their consumption is not dependent on caloric or sensory properties of ethanol, and (3) they exhibit a relapse-like effect (Devineni and Heberlein 2009). The conditioned preference assay further demonstrates that flies find ethanol intoxication rewarding (Kaun et al. 2011). In both assays flies were willing to overcome negative stimuli (bitter-tasting compound or electric shock, respectively) in order to obtain ethanol or the ethanol-associated cue, suggesting compulsive-like behavior toward ethanol.

However, certain important criteria for addiction have not yet been met in flies. For example, it has not been shown that voluntary ethanol consumption leads to ethanol tolerance, that ethanol removal causes withdrawal symptoms, or that flies are willing to “work” in order to obtain ethanol. The latter criterion could be demonstrated by showing that flies exhibit operant responding for ethanol, a paradigm that has not yet been developed in *Drosophila*.

In addition to developing new and more complex behavioral assays, many questions remain to be tested using the existing assays for ethanol consumption and reward. For example, flies are typically trained in the conditioned preference assay for only 1 day; it is unknown whether this preference would be altered if flies were trained intermittently over many days. Preliminary data suggest that such long-term ethanol exposures are still perceived as rewarding (K. Kaun and U. Heberlein, unpublished data). In addition, it is unknown whether flies in the conditioned preference assay show behavior indicative of a relapse-like effect, which could be demonstrated by showing that flies exhibit reinstatement to the ethanol-associated cue following extinction.

While more complex assays improve the validity of *Drosophila* as a model system to study drug addiction, these assays are necessarily more cumbersome and time-consuming, thus making large-scale genetic screening difficult. It is therefore important to understand how the simple and more complex assays for drug-induced behavior are related. Evidence in humans and rodent models is generally consistent with the notion that resistance to the acute effects of ethanol predicts increased ethanol consumption and risk of abuse (Schuckit 1994; Schuckit and Smith 1996; Morean and Corbin 2010). While this association suggests that an overlapping set of genes regulates both simple and complex drug behaviors, several exceptions to this correlation exist (e.g. Colombo et al. 2000; Phillips et al. 1998; Boehm et al. 2004), indicating that factors other than acute drug sensitivity modulate drug intake.

A recent study analyzed the relationships between initial ethanol sensitivity, ethanol tolerance, and voluntary ethanol consumption in flies. Ethanol consumption was positively correlated with the development of tolerance, but not with naive sensitivity to the sedating or hyperactivating effects of ethanol (Devineni et al. 2011). These results suggest that complex behaviors such as voluntary ethanol consumption are not simply readouts of acute responses; they likely incorporate acute sensitivity and the development of tolerance in addition to other factors, such as experience and learning. Some genes have been found to regulate multiple ethanol-induced behaviors: for example *kra* regulates sedation sensitivity, tolerance, and voluntary ethanol consumption (Berger et al. 2008; Devineni and Heberlein 2009). In contrast, other genes regulate individual ethanol-induced behaviors: for instance *sca* mediates conditioned ethanol preference, but not acute ethanol sensitivity (Kaun et al. 2011; LaFerriere et al. 2008). Thus, while the simpler assays are ideal for rapidly identifying ethanol-related genes and have some predictive value for more complex behaviors, the development of high-throughput versions of the more complex assays will be necessary in order to apply the power of *Drosophila* genetics to the study of addiction-like behaviors.

Intriguingly, forward genetic screens in flies have identified many different and unexpected molecular mechanisms that regulate ethanol-related behavior. These mechanisms include signaling pathways such as the cAMP and PI3K pathways that have broad roles in regulating many diverse processes such as development, cell signaling, and neuronal plasticity. Many of the signaling pathways that have been identified can affect each other in complex ways, so it is likely that some pathways may have a more direct role in regulating ethanol responses than others. In most cases it is unknown whether the molecules that have been implicated are direct targets of ethanol or are required downstream of ethanol binding.

One theme that emerges from this large number of pathways is the involvement of molecules affecting molecular or cellular plasticity (e.g. cytoskeletal regulators, ion channels, synaptic molecules), which appear to be recruited to induce behavioral changes in response to ethanol. Much work remains to be done in uncovering how different mechanisms interact to regulate ethanol-related behavior, and how the molecular and cellular changes induced by acute ethanol exposure are translated into addiction-like responses. For example, cytoskeletal changes caused by acute ethanol exposure may be required to induce changes in synapse number, which consequently may mediate ethanol tolerance and preference behaviors. Understanding the relationship between mechanisms mediating acute and long-term responses to ethanol is key to understanding the addictive properties of the drug. The fly is ideally suited to this task due to the availability of tools to investigate these mechanisms with high spatial and temporal resolution.

The next decade should witness the discovery of many novel mechanisms underlying addiction-related behaviors in flies as the number of tools available to study molecular and neural processes is expanding at a rapid rate. Based on what we have learned in the last 15 years from *Drosophila* addiction research, we expect that these novel mechanisms will be relevant to mammalian models and provide novel targets for the development of pharmacotherapies for drug addiction.
